# Dengue Sentinel Traveler Surveillance: Monthly and Yearly Notification Trends among Japanese Travelers, 2006–2014

**DOI:** 10.1371/journal.pntd.0004924

**Published:** 2016-08-19

**Authors:** Munehisa Fukusumi, Takeshi Arashiro, Yuzo Arima, Tamano Matsui, Tomoe Shimada, Hitomi Kinoshita, Ashley Arashiro, Tomohiko Takasaki, Tomimasa Sunagawa, Kazunori Oishi

**Affiliations:** 1 Infectious Disease Surveillance Center, National Institute of Infectious Diseases, Tokyo, Japan; 2 Osaka University Graduate School of Medicine, Osaka, Japan; 3 School of Medicine, Tokyo Medical and Dental University, Tokyo, Japan; 4 Department of Virology 1st, National Institute of Infectious Diseases, Tokyo, Japan; 5 Kanagawa Prefectural Institute of Public Health, Chigasaki, Japan; Duke-NUS GMS, SINGAPORE

## Abstract

**Background:**

Dengue is becoming an increasing threat to non-endemic countries. In Japan, the reported number of imported cases has been rising, and the first domestic dengue outbreak in nearly 70 years was confirmed in 2014, highlighting the need for greater situational awareness and better-informed risk assessment.

**Methods:**

Using national disease surveillance data and publically available traveler statistics, we compared monthly and yearly trends in the destination country-specific dengue notification rate per 100,000 Japanese travelers with those of domestic dengue cases in the respective country visited during 2006–2014. Comparisons were made for countries accounting for the majority of importations; yearly comparisons were restricted to countries where respective national surveillance data were publicly available.

**Results:**

There were 1007 imported Japanese dengue cases (Bali, Indonesia (n = 202), the Philippines (n = 230), Thailand (n = 160), and India (n = 152)). Consistent with historic local dengue seasonality, monthly notification rate among travelers peaked in August in Thailand, September in the Philippines, and in Bali during April with a smaller peak in August. While the number of travelers to Bali was greatest in August, the notification rate was highest in April. Annually, trends in the notification rate among travelers to the Philippines and Thailand also closely reflected local notification trends.

**Conclusion:**

Travelers to dengue-endemic countries appear to serve as reliable “sentinels”, with the trends in estimated risk of dengue infection among Japanese travelers closely reflecting local dengue trends, both seasonally and annually. Sentinel traveler surveillance can contribute to evidence-based pretravel advice, and help inform risk assessments and decision-making for importation and potentially for subsequent secondary transmission. As our approach takes advantage of traveler data that are readily available as a proxy denominator, sentinel traveler surveillance can be a practical surveillance tool that other countries could consider for implementation.

## Author Summary

With increasing globalization, the threat of dengue is rising in areas that were previously unaffected. Japan has been experiencing a rise in notifications of imported cases, and in 2014 confirmed the first domestic outbreak in nearly 70 years. Such events prompted the country to more actively utilize existing imported dengue case data among travelers to inform situational awareness, risk assessment, and evidence-based decision-making. Using both national disease surveillance data and publically available traveler statistics, we compared monthly and yearly trends between reported numbers of dengue cases among Japanese travelers and those of domestic dengue cases in the countries visited. By using the number of Japanese travelers to a dengue-endemic country as an approximate denominator, we estimated the risk of dengue infection among travelers to the country. This method is more appropriate than simply monitoring the number of reported imported cases because it accounts for fluctuating numbers of travelers, such as during vacation periods. This study demonstrated that the trends in dengue notifications among travelers were consistent with local dengue trends, both yearly and seasonally. Our simple approach, which takes advantage of existing data, may be readily adopted elsewhere to help inform risk of importation and potential subsequent domestic transmission.

## Introduction

Dengue is a mosquito-borne febrile viral disease that is endemic in over 100 countries in tropical and subtropical regions with notifications increasing 30-fold over the last 50 years, affecting an estimated 100 million people annually [1–3]. Over these decades, it has become apparent that dengue shows fairly consistent seasonality with periodic epidemic years in many endemic areas [4–5].

Importantly, dengue is becoming an emerging threat to non-endemic countries. The number of people traveling to dengue-endemic areas has been rising, along with reports of dengue cases among travelers. In fact, dengue is currently the second most common specific etiologic diagnosis among returned travelers with febrile illness after malaria globally, and the most common febrile illness among travelers returning from Southeast Asia and South Central Asia [6–8]. Imported dengue cases can contribute to further spread of dengue in non-endemic areas when competent vectors such as *Aedes albopictus* and *Ae*. *aegypti* are present. In recent years, autochthonous dengue outbreaks following importation have been reported in several non-endemic countries such as France, Croatia, Portugal, and the United States [9–12].

Japan has been no exception. While dengue is not endemic in Japan, approximately 50 to 200 imported cases have been reported annually in the last 10 years with an upward trend, causing growing concern of domestic spread as *Ae*. *albopictus* is active in much of Japan from spring to fall [13]. Such concern became a reality in summer 2013, when a German dengue case suspected to have been infected while traveling in Japan was reported [14], and in 2014 Japan experienced the first confirmed domestic dengue outbreak in nearly 70 years with 162 autochthonous cases [15,16]. The autochthonous dengue strain was shown to be similar to those circulating in Southeast Asia, the region associated with the majority of Japanese imported cases [15].

This recent chain of events of autochthonous dengue transmission in Japan, arising from importation of dengue, highlighted the need for greater situational awareness, risk assessment, and evidence-based communication. Since the domestic outbreak of 2014, interest in situational awareness of dengue in endemic areas frequented by travelers has increased, and such information, together with existing sentinel traveler case data, may feed into better informed risk assessment. Previously, Nakamura et al. demonstrated that the risk of dengue among Japanese travelers appeared to be greater during historic dengue high season relative to low season in the endemic countries that they had visited; these findings supported the idea that the local dengue situation may directly affect the risk of infection among travelers [13]. However, the association between yearly trends in imported cases and yearly domestic fluctuations in the associated destination countries was not assessed, although periodic epidemic years occur in many endemic areas [4,5]. Such assessment became increasingly important after the domestic outbreak—if yearly trends in endemic destination areas are correlated with those among travelers, then the former can directly inform risk assessment for secondary domestic transmission following importation, assuming that a higher number of imported cases would increase the risk of secondary transmission and accounting for activity level of competent mosquitoes.

Therefore, building on our previous work and the experience from others [5–8], our objectives were to describe and compare the trends in notifications between sentinel traveler dengue cases among Japanese travelers with those of domestic dengue cases in the country visited, by month and by year. The monthly assessment updates the seasonality findings reported by Nakamura et al. [13], while the yearly assessment approach highlights additional benefits from sentinel traveler surveillance.

## Methods

### Data sources for imported dengue cases in Japan

Dengue has been a notifiable disease in Japan since April 1999. Physicians are required to report demographic, clinical and exposure history information of laboratory-confirmed cases through the Japanese national infectious disease surveillance system (National Epidemiological Surveillance of Infectious Diseases (NESID)). To report a dengue case in Japan, at least one of the following laboratory confirmation methods is required: 1) virus isolation; 2) detection of virus-specific nucleic acid sequences by polymerase chain reaction (PCR) method; 3) detection of dengue nonstructural protein 1 (NS1) antigen in serum; or 4) detection of anti-dengue IgM antibody in serum; or seroconversion or 4-fold rise in IgG or IgM antibody titers in paired serum samples in neutralization test or hemagglutination inhibition test [13,16].

Dengue cases reported during 2006–2014 were extracted from NESID. Cases were excluded from analyses if any of the following criteria were met: 1) missing information on country traveled; 2) traveled to multiple countries during the potential incubation period (2–14 days); 3) home address outside of Japan (i.e., potentially not included in the proxy denominator of Japanese travelers); 4) known to be a non-Japanese national (i.e., potentially not included in the proxy denominator of Japanese travelers); or 5) traveled to a dengue-endemic country that had a total of 30 or fewer reported imported cases during the nine-year study period (due to sample size constraints).

Comparisons of temporal trends in notifications between sentinel traveler dengue cases among Japanese travelers and domestic dengue cases in the country visited were made for India, Indonesia, the Philippines, and Thailand because these countries accounted for the majority of imported dengue cases to Japan.

### Data sources for Japanese travelers

Annual international traveler data for Japanese travelers that visited India, Indonesia, the Philippines, and Thailand were obtained from the Japan National Tourist Organization (http://www.jnto.go.jp/jpn/reference/tourism_data/departure_trends/index.html) [17]. Data for Japanese travelers visiting India and Indonesia were restricted to those from 2006–2013, as 2014 data were not yet available as of July 2015. Japanese travelers to the Philippines and Indonesia were based on Japanese residency rather than Japanese nationality, as the latter were unavailable.

Monthly traveler data for Japanese travelers that visited Bali province, Indonesia, the Philippines, and Thailand were obtained from the Japanese Tourism Marketing Company (http://www.tourism.jp/statistics/outbound/) [18]. For Indonesia, monthly traveler data were only available for Bali province. Philippines data were restricted to those from 2006–2013, as monthly traveler data were not yet available for 2014 as of July 2015. Monthly Japanese traveler data were not available for India.

### Data sources for domestic dengue cases in select endemic countries

For dengue cases reported domestically in the select endemic countries, annually reported numbers of dengue cases for the Philippines and Thailand were available and obtained from the World Health Organization’s Regional Office for the Western Pacific’s dengue website (http://www.wpro.who.int/topics/dengue/en/) [19] and the national surveillance reports for dengue published by the Bureau of Epidemiology, Department of Disease Control Ministry of Public Health, Thailand (http://www.boe.moph.go.th/) [20], respectively.

### Data analysis

Reported imported dengue cases in Japan were described by age, sex, and country visited. To compare the country-specific trends in imported dengue cases among Japanese travelers with the number of cases reported in the country visited 1) by month and 2) by year, the following methods were used:

1. Country-specific monthly notification rate: The number of reported imported Japanese dengue cases who returned from a dengue-endemic country (India, the Philippines, and Thailand) or area (Bali province, Indonesia) for each month was aggregated over the period of 2006–2014. Monthly data were summed over these years as the number of cases reported for each month was small and the monthly distribution remained consistent over the years. Similarly, the total number of Japanese travelers who visited the country/area for each calendar month was aggregated over the period of 2006–2014. Then, the notification rate was calculated by dividing the above aggregated number of dengue cases by the aggregated number of Japanese travelers who visited the country/area.
2. Country-specific yearly notification rate: To calculate the notification rate of dengue infections among Japanese travelers who returned from a dengue-endemic country (India, Indonesia, the Philippines, and Thailand), the number of reported imported Japanese dengue cases who returned from a dengue-endemic country was divided by the total number of Japanese travelers who visited the country in the same year. The country-specific yearly notification rate trend among Japanese travelers was compared with the yearly trend in the total number of dengue cases reported domestically in the respective country visited. Yearly comparisons were restricted to the Philippines and Thailand where respective national surveillance data were publicly available.

### Ethics statement

Only the extracted NESID data contained individual level data, which did not include identifiable information. None of the other data sources included individual unit data. Thus, ethical approval and informed consent were not required.

## Results

### Description of reported imported dengue cases in Japan

**Fig 1:**
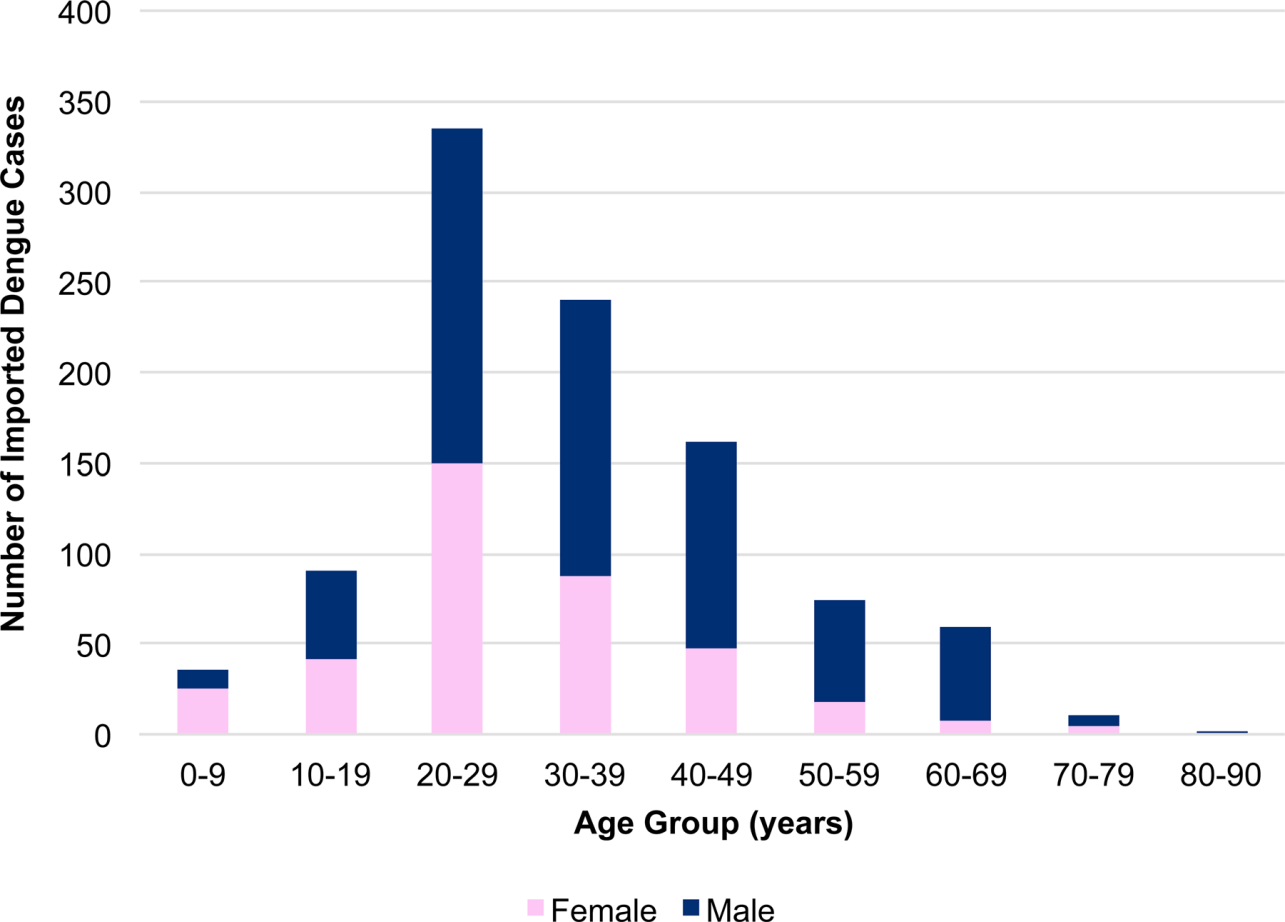
Distribution of reported imported dengue cases by sex and age, Japan, 2006–2014 (n = 1007).

**Fig 2:**
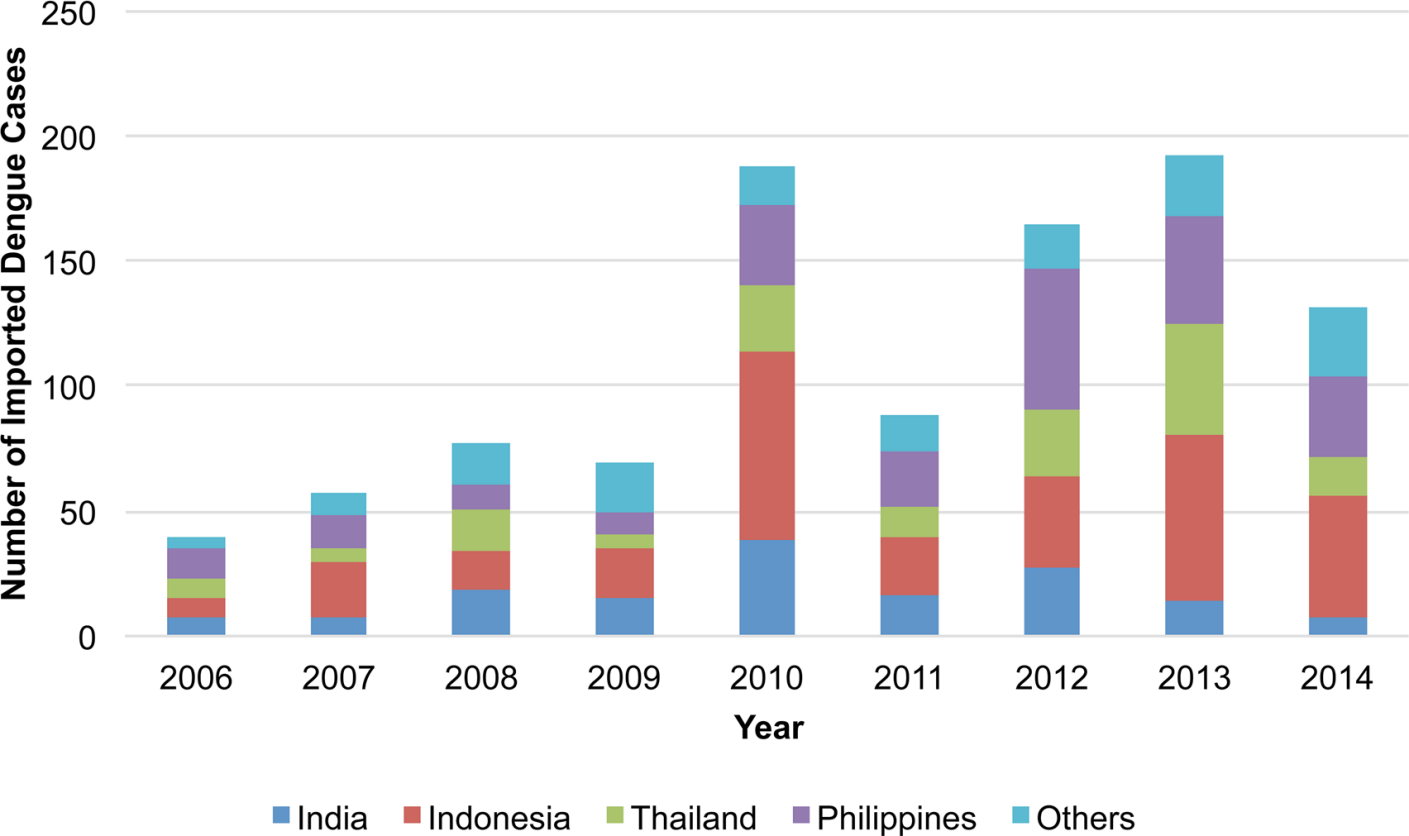
Number of reported imported dengue cases by year and country visited, Japan, 2006–2014 (n = 1007).

A total of 1512 dengue cases were reported during the study period from 2006–2014. 162 were autochthonous cases associated with the domestic outbreak in 2014, while 343 cases were excluded based on the exclusion criteria (including 95 (6%) cases excluded for visiting multiple countries during the potential incubation period). Thus, a total of 1007 (67%) cases remained for analysis. Among those, 625 (62%) were male and the median age was 31 years (range: 0–90). For both sexes, the age group most reported was 20–29 years (326/1007 [32%]) (Fig 1). The countries associated with the largest number of imported dengue cases were Indonesia (n = 317 [31%]), the Philippines (n = 230 [23%]), Thailand (n = 160 [16%]), and India (n = 152 [15%]), comprising approximately 85% of the 1007 cases (Fig 2).

### Monthly notification trends

**Fig 3:**
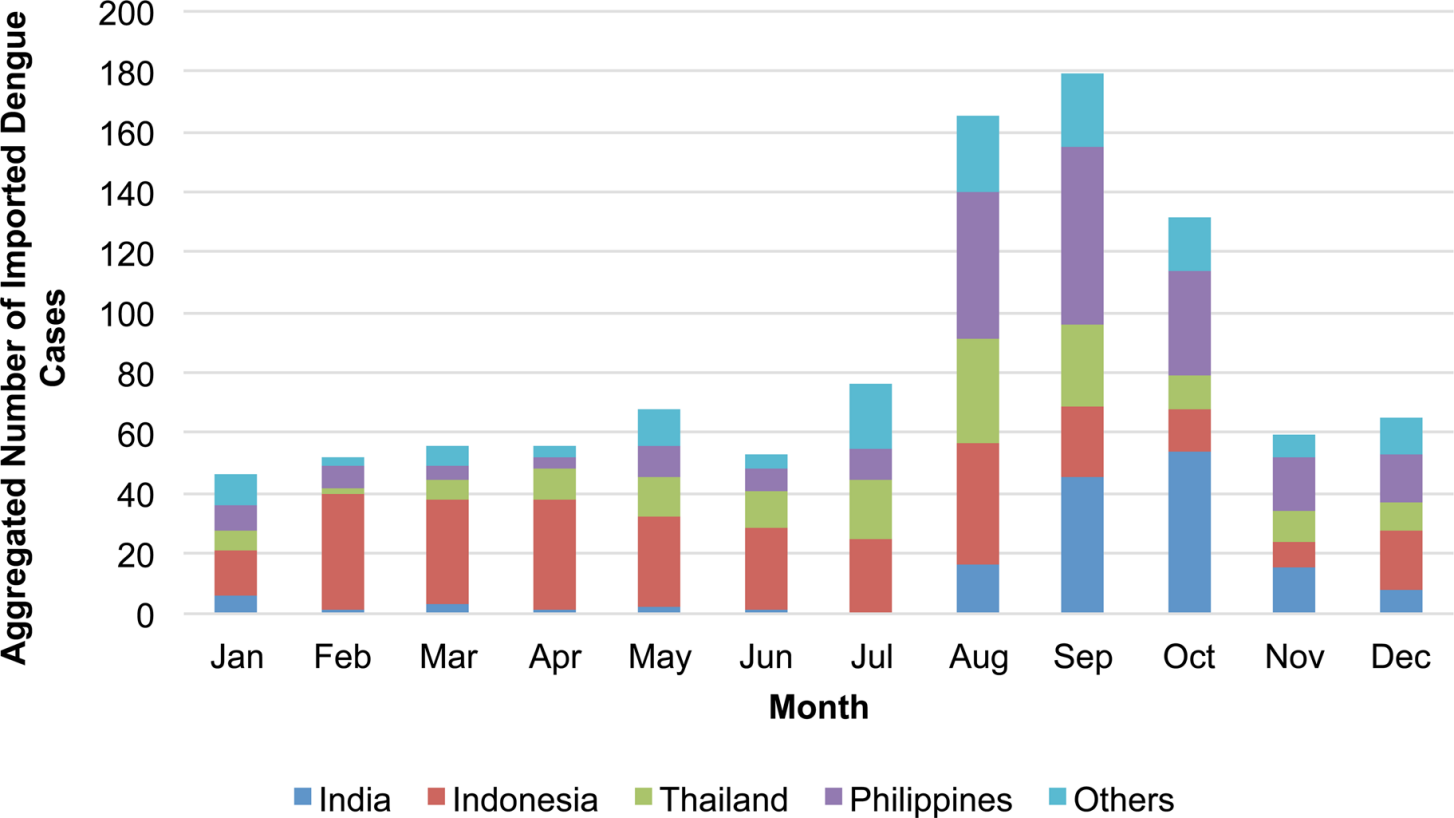
Number of reported imported dengue cases aggregated over month by country visited, Japan, 2006–2014 (n = 1007).

The monthly number of reported imported cases from 2006–2014 was highest during August through October, although the distribution varied by country. For example, the number of cases associated with visiting Thailand peaked in August, while the Philippines peaked in September and India was highest in October (Fig 3; Table 1). In Indonesia, the highest numbers of cases were observed during February to April and in August (Fig 3); similarly, among those that visited Bali province, Indonesia, the trend was bimodal, peaking during February to April and again in August (Table 1).

**Table 1:** Numbers of reported imported dengue cases, Japanese travelers, and reported dengue cases per 100,000 Japanese travelers by country visited and month, 2006–2014.

	No. dengue cases imported to Japan			No. Japanese travelers			No. dengue cases per 100,000 Japanese travelers		
	Bali	Thailand	Philippines (2006–2013)*	Bali	Thailand	Philippines (2006–2013)*	Bali	Thailand	Philippines (2006–2013)*
**Jan**	8	7	6	174364	1007109	267870	4.59	0.70	2.24
**Feb**	25	2	7	198189	1019907	266300	12.61	0.20	2.63
**Mar**	24	6	4	196709	988660	281578	12.20	0.61	1.42
**Apr**	28	10	3	163651	840282	251202	17.11	1.19	1.19
**May**	19	13	11	154592	731768	227381	12.29	1.78	4.84
**Jun**	20	12	6	186919	755825	220943	10.70	1.59	2.72
**Jul**	12	19	11	215693	862648	257898	5.56	2.20	4.27
**Aug**	26	34	43	245719	1124253	316896	10.58	3.02	13.57
**Sep**	12	27	48	239311	1005660	263725	5.01	2.68	18.20
**Oct**	8	11	32	202966	819005	230490	3.94	1.34	13.88
**Nov**	7	10	14	177721	928853	237296	3.94	1.08	5.90
**Dec**	13	9	13	171623	967048	259946	7.57	0.93	5.00

Data for Japanese travelers visiting the Philippines by month were not available for 2014.

**Fig 4:**
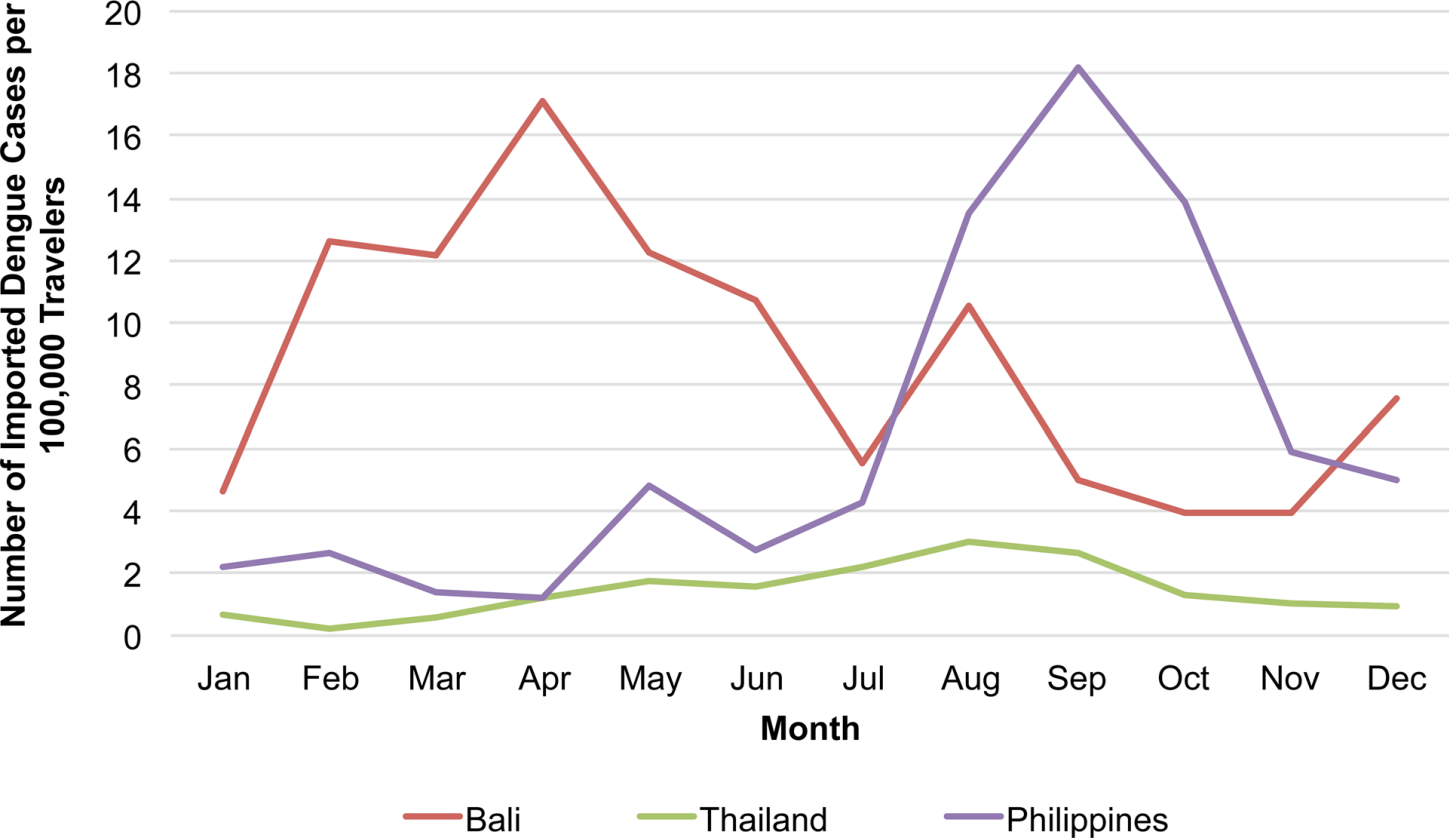
Number of reported imported dengue cases per 100,000 travelers aggregated over month by country visited, Japan, 2006–2014. For Indonesia, monthly data for Japanese travelers were only available for Bali province. Philippines data were restricted to those from 2006–2013 as monthly traveler data were not available for 2014. Monthly Japanese traveler data were not available for India.

When the monthly number of imported cases from 2006–2014 was calculated as the monthly notification of country-specific imported cases per 100,000 Japanese travelers, the overall monthly trend was similar to the number of imported cases (Fig 4; Table 1). Notification rates for Thailand and the Philippines peaked in the same months as the number of imported cases. For Bali, the notification rate was highest during February to May, peaking in April, and with a smaller peak in August. While the number of travelers to Bali was greatest in August, the notification rate was highest in April (Table 1).

### Yearly notification trends

**Fig 5:**
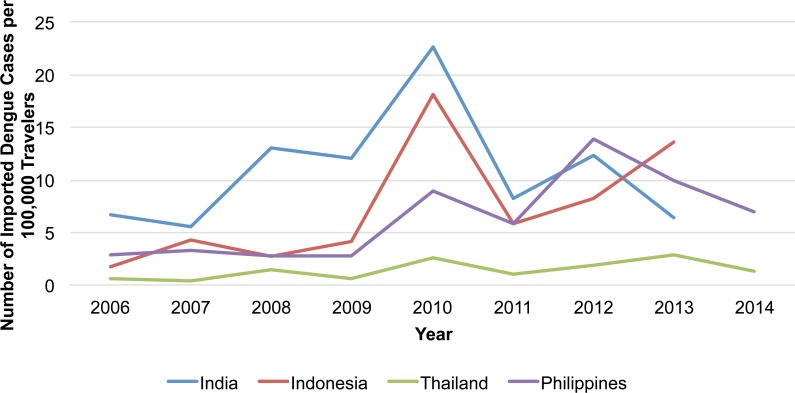
Number of reported imported dengue cases per 100,000 travelers by year and country visited, Japan, 2006–2014. Data for Japanese travelers visiting India and Indonesia were not available for 2014.

**Fig 6:**
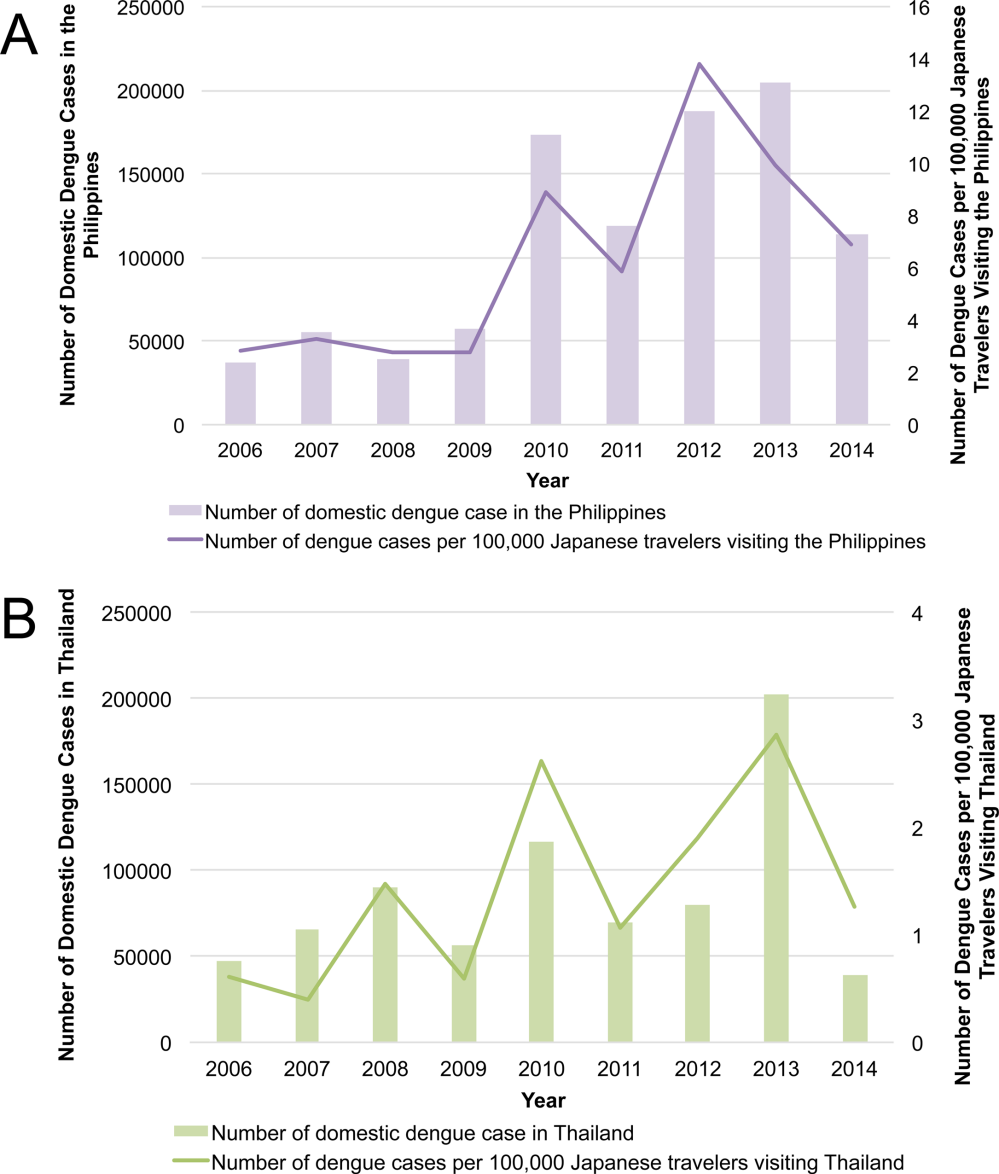
Number of domestic dengue cases reported from (A) the Philippines and (B) Thailand, and the annual number of imported dengue cases per 100,000 travelers visiting each country, 2006–2014.

The annual number of reported imported cases from 2006–2014 was highest in 2013 (n = 192), followed by 2010 (n = 188). Although the distribution varied by country visited, there was an increase from 2009 to 2010 followed by a decrease in 2011 for all four countries (Fig 2). When calculated as the annual country-specific notification rate (i.e., estimated from number of reported imported cases per 100,000 Japanese travelers per year), differences by country visited and by year were observed. For instance, the annual notification rate for Thailand was consistently lower than those of the other three countries. Notably, rates among travelers also saw an increase from 2009 to 2010 followed by a decrease in 2011 for all four countries (Fig 5). The trend in the annual number of dengue cases per 100,000 travelers visiting the Philippines closely mirrored the trend in the annual number of dengue cases reported in the Philippines (Fig 6A). Similarly, the trend in the annual number of dengue cases per 100,000 travelers visiting Thailand reflected the annual number of dengue cases reported in Thailand (Fig 6B).

## Discussion

In the present study, we found that travelers to dengue-endemic countries appear to serve as “sentinels”. Based on retrospective analysis of national Japanese surveillance data from 2006–2014, the estimated risk of dengue infection among Japanese travelers closely reflected local dengue trends, seasonally and annually.

The overall trend in Japanese dengue traveler cases was the same as previously reported [13]. Male cases remained dominant, with case distribution skewed towards young adults, although the age distribution of travelers is unknown. Moreover, despite yearly variation, the majority continued to be associated with travel to India, Indonesia, Thailand, and the Philippines, comprising 70–90% of imported cases.

Similarly, as previously reported [13], country-specific seasonality in notification rate among Japanese travelers was consistent with historic dengue seasonality in the endemic countries visited. For instance, the notification rate among travelers to Thailand and the Philippines was highest during July to September, coinciding with their historic dengue high seasons [4,13,21]. For Bali province, an area popular for Japanese tourists, we found that the notification rate among travelers peaked in April, which corresponds to local peak activity during 2001–2010, ranging from February to June [22].

We also found that the yearly country-specific dengue notification rate among travelers was correlated with the yearly number of reported dengue cases among the respective dengue-endemic countries. In endemic countries, dengue epidemics are known to occur every few years [4,5], and notifications among travelers mirrored such trends. The year 2010 was particularly notable, as an increase was observed in notification rates among travelers, domestically in multiple endemic countries, and in the entire Western Pacific Region [4,22].

Similar correlations between trends in imported cases and local trends in dengue-endemic countries have been reported previously. For example, patterns of local incidence of dengue in the Pacific Islands closely reflected patterns of incidence of dengue imported from these areas to New Zealand [23]. The international GeoSentinel Surveillance Network suggested that seasonality of dengue in travelers is similar to that in the local population, and thus such data may benefit those advising prospective travelers or those diagnosing ill-returned travelers [5]. Additionally, an excess of cases among travelers to Southeast Asia in the years 1998 and 2002 was shown to reflect epidemics in Southeast Asia. Thus, the concept of sentinel traveler surveillance is not new; various approaches and networks on several diseases have been established, particularly in Europe and North America (e.g., TropNet Europe, GeoSentinel, Canadian Travel Medicine Network) [6–8, 24–26]. However, existing systems have been mostly limited to participating private clinics and trend assessment of sentinel cases has often been based on number of cases or proportionate morbidity, using ill travelers as the denominator. Interpretation of trends based on numerators or proportionate morbidity alone requires caution, as they may not reflect incidence. More rigorous methods have gone further to estimate person-time incidence among cohorts of travelers [27]; however, such special studies can be costly and time-consuming.

The strength of our method is that we use publicly available “big data” as a proxy to estimate country-specific dengue incidence among travelers (strictly speaking a ratio, as crudely comparing number of cases to the number of travelers to a given location and period). Inferring trends from the frequency of imported cases alone requires caution, as risk cannot be determined solely from numerator data. As with Bali, while there was a high number of reported cases in August, the number of Japanese travelers tended to be highest in August (summer vacation season), and the estimated risk among travelers to Bali was higher from February to May (Table 1).

It is important to consider separately the risk of infection for travelers and the potential for secondary transmission following importation. While the risk for travelers to Bali may be highest from February to May, risk of further transmission in Japan is minimal from such importations, as the vector is not active during these months in most of Japan. On the contrary, importation of viremic cases during the summer months, even those that are asymptomatic [28], would indicate a higher risk of secondary transmission and a greater public health concern, as the vector is active and more people are outside with more exposed skin. Additionally, all else being equal, given our findings on annual trends, the risk of importation—and potentially subsequent secondary transmission—may be higher when endemic countries are experiencing an epidemic year. Our approach enables evidence-based risk communication to travelers and help inform risk assessment and decision-making for secondary transmission.

Furthermore, sentinel traveler surveillance may provide potentially important information to endemic countries, especially low-capacity/resource-limited countries. In such settings, surveillance for dengue may be limited, and information from sentinel traveler surveillance from import countries may provide earlier awareness [5, 23]. Additionally, if virologic data such as genotype are lacking or unavailable in a timely fashion in the endemic country, such information may potentially be of value for the endemic country.

There are important limitations in this study. Specific human activity/behavior has been reported as major risk factors for dengue infection [5,29–32], but specific travel locations or activity/behavior at destination sites were not available from surveillance data. These aspects may confound the association between country visited and risk of dengue infection. The difference in notification rates among the destination countries could be explained by differing behaviors of Japanese travelers visiting those countries that directly relate to dengue infection risk, and such inter-country comparisons require caution. In addition, as dengue activity has been shown to vary at the local level [22,30,33–35], the risk of infection in areas travelers visit may differ from those that are frequented by the local population [36]. Nevertheless, in our study, the monthly and yearly notification rate among sentinel travelers correlated closely with trends observed from national surveillance of the endemic countries. Secondly, the risk of dengue infection for Japanese travelers might be underestimated, as the information is based on reported surveillance data from patients who sought healthcare [36]. Also, as dengue has a short incubation period, many Japanese who developed illness abroad may be missed. However, as such bias likely remains consistent over time, it is unlikely to influence the interpretation of temporal trends observed in sentinel traveler surveillance. Similarly, while our assessments were based on a few select countries (limited to those comprising the majority of Japanese sentinel traveler cases), given sufficient sample sizes, we do not believe that the correlations observed would only apply to these specific countries but not to others. Lastly, 316 (31%) of the cases were confirmed by detection of anti-dengue IgM antibody in serum from a single specimen, and this may pose a concern as other flavivirus (e.g. yellow fever, Japanese encephalitis, Zika viruses) infections could result in false detections. However, as a sensitivity analysis restricted to cases confirmed by methods other than IgM resulted in very similar yearly and monthly trends, our interpretations remain qualitatively unchanged (see Supporting Information). Given that our approach takes advantage of readily available data that are often available at little to no cost, we encourage others to explore sentinel traveler surveillance based on their surveillance and traveler data and corroborate our findings.

To conclude, we found that trends in dengue notifications among Japanese travelers closely reflected local dengue trends, both seasonally and annually. Sentinel traveler surveillance can be a practical, low-cost, evidence-based tool to help inform risk assessment, situational awareness, and decision-making, in the effort to both reduce infection among travelers and the potential for secondary domestic transmission. Enhanced awareness among travelers and healthcare providers, as well as global information-sharing, will continue to be essential for combating the dengue threat. Given the ever-increasing expansion of dengue and other vector-borne diseases, maximizing the utilization of sentinel traveler surveillance is more important than ever.

## Supporting Information

S1 Checklist STROBE checklist. (DOC)

S1 File Supporting Excel files for Figs 1–6. (XLSX)

S2 File Supporting Excel file for analysis restricted to non-IgM methods. (XLSX)
