# Supplementary material for: Dengue Sentinel Traveler Surveillance: Monthly and Yearly Notification Trends among Japanese Travelers, 2006–2014
Source: PLoS Negl Trop Dis. 2016 Aug 19;10(8):e0004924. doi: 10.1371/journal.pntd.0004924 (PMC4991785; doi:10.1371/journal.pntd.0004924)
Supplement: S1 Checklist — (DOC) [file pntd.0004924.s001.doc]

STROBE Statement—checklist of items that should be included in reports of observational studies

|  | Item No | Recommendation |
| --- | --- | --- |
| **Title and abstract** | 1 | (*a*) Indicate the study’s design with a commonly used term in the title or the abstract　(Study’s design is description of notified surveillance data and described in both the title and the abstract) |
|  |  | (*b*) Provide in the abstract an informative and balanced summary of what was done and what was found  (Abstract provides a clear summary of descriptive approach used and the monthly and yearly results) |
| Introduction | | |
| Background/rationale | 2 | Explain the scientific background and rationale for the investigation being reported (Background of increasing importations and further transmissions in non-endemic settings is explained along with rationale to make best use of existing data) |
| Objectives | 3 | State specific objectives, including any prespecified hypotheses  (Given background and rationale, specific objectives (e.g. knowledge gap regarding annual trends) explicitly described in final paragraph of Introduction) |
| Methods | | |
| Study design | 4 | Present key elements of study design early in the paper  (Source of data and approach of comparing temporal trends between sentinel traveler cases and countries visited described in first 2 pages of Methods) |
| Setting | 5 | Describe the setting, locations, and relevant dates, including periods of recruitment, exposure, follow-up, and data collection  (Details regarding data sources and extraction and study period described in first 3 pages of Methods. As all data are from passive surveillance or publicly available traveler data, no recruitment or follow-up) |
| Participants | 6 | (“Participants” were those passively reported through notifiable surveillance system, as described in first page of Methods. All other data were aggregate data (i.e. annual no. cases reported from surveillance systems and traveler statistics). Study was descriptive case-series exploring temporal trends, accounting for number of travelers to an at-risk location as a proxy denominator).  (*a*) *Cohort study*—Give the eligibility criteria, and the sources and methods of selection of participants. Describe methods of follow-up  *Case-control study*—Give the eligibility criteria, and the sources and methods of case ascertainment and control selection. Give the rationale for the choice of cases and controls  *Cross-sectional study*—Give the eligibility criteria, and the sources and methods of selection of participants |
|  |  | (*b*) *Cohort study*—For matched studies, give matching criteria and number of exposed and unexposed  *Case-control study*—For matched studies, give matching criteria and the number of controls per case |
| Variables | 7 | Clearly define all outcomes, exposures, predictors, potential confounders, and effect modifiers. Give diagnostic criteria, if applicable  (No comparison group and no formal statistical testing conducted to analyze association between outcomes and exposures/predictors, confounders, or effect modifiers. Diagnostic and exclusion criteria were fully described in paragraphs 1-2 of Methods.). |
| Data sources/ measurement | 8* | For each variable of interest, give sources of data and details of methods of assessment (measurement). Describe comparability of assessment methods if there is more than one group  (Sources of data and methods of measurement described in pages 1-3 of Methods; no comparison group) |
| Bias | 9 | Describe any efforts to address potential sources of bias  (Exclusion criteria to reduce potential sources of bias described in first 2 pages of Methods) |
| Study size | 10 | Explain how the study size was arrived at  (No formal sample size calculations performed since no sampling or formal statistical testing performed; however countries with few cases (<=30 cases total) excluded as described in exclusion criteria) |
| Quantitative variables | 11 | Explain how quantitative variables were handled in the analyses. If applicable, describe which groupings were chosen and why  (N/A; only quantitative variable was age and not part of main analysis) |
| Statistical methods | 12 | (*a*) Describe all statistical methods, including those used to control for confounding  (All statistical methods described under “Data analysis”) |
|  |  | (*b*) Describe any methods used to examine subgroups and interactions  (Sub-analysis based on restriction discussed briefly in Discussion) |
|  |  | (*c*) Explain how missing data were addressed  (Cases with missing data excluded based on defined exclusion criteria, with full disclosure of those excluded in Results) |
|  |  | (*d*) *Cohort study*—If applicable, explain how loss to follow-up was addressed  *Case-control study*—If applicable, explain how matching of cases and controls was addressed  *Cross-sectional study*—If applicable, describe analytical methods taking account of sampling strategy |
|  |  | (*e*) Describe any sensitivity analyses  (Sub-analysis based on restriction discussed briefly in Discussion) |

Continued on next page

| Results | | |
| --- | --- | --- |
| Participants | 13* | (a) Report numbers of individuals at each stage of study—eg numbers potentially eligible, examined for eligibility, confirmed eligible, included in the study, completing follow-up, and analysed  (Cases excluded due to exclusion criteria fully disclosed in first paragraph of Results) |
|  |  | (b) Give reasons for non-participation at each stage  (NA) |
|  |  | (c) Consider use of a flow diagram  (NA as no active recruitment or follow-up process) |
| Descriptive data | 14* | (a) Give characteristics of study participants (eg demographic, clinical, social) and information on exposures and potential confounders  (Limited participant characteristics, but described in first paragraph of Results and Figure 1) |
|  |  | (b) Indicate number of participants with missing data for each variable of interest  (First paragraph of Results) |
|  |  | (c) *Cohort study*—Summarise follow-up time (eg, average and total amount) |
| Outcome data | 15* | *Cohort study*—Report numbers of outcome events or summary measures over time |
|  |  | *Case-control study—*Report numbers in each exposure category, or summary measures of exposure |
|  |  | *Cross-sectional study—*Report numbers of outcome events or summary measures |
| Main results | 16 | (Main results displayed in Figs 2- 6. Crude numbers disclosed in Table 1)  (*a*) Give unadjusted estimates and, if applicable, confounder-adjusted estimates and their precision (eg, 95% confidence interval). Make clear which confounders were adjusted for and why they were included |
|  |  | (*b*) Report category boundaries when continuous variables were categorized |
|  |  | (*c*) If relevant, consider translating estimates of relative risk into absolute risk for a meaningful time period |
| Other analyses | 17 | Report other analyses done—eg analyses of subgroups and interactions, and sensitivity analyses  (Sub-analysis based on restriction discussed briefly in Discussion) |
| Discussion | | |
| Key results | 18 | Summarise key results with reference to study objectives  (First 2 pages of Discussion) |
| Limitations | 19 | Discuss limitations of the study, taking into account sources of potential bias or imprecision. Discuss both direction and magnitude of any potential bias  (Last 3 pages of Discussion discuss limitations, including direction and magnitude of potential bias) |
| Interpretation | 20 | Give a cautious overall interpretation of results considering objectives, limitations, multiplicity of analyses, results from similar studies, and other relevant evidence  (Pg. 3 onwards of Discussion discuss interpretations, limitations, subanalysis, and results from other studies) |
| Generalisability | 21 | Discuss the generalisability (external validity) of the study results  (Discussed in the limitations and call to corroborate findings) |
| Other information | | |
| Funding | 22 | Give the source of funding and the role of the funders for the present study and, if applicable, for the original study on which the present article is based  (Disclosed in the journal’s Financial Disclosure section) |

*Give information separately for cases and controls in case-control studies and, if applicable, for exposed and unexposed groups in cohort and cross-sectional studies.

**Note:** An Explanation and Elaboration article discusses each checklist item and gives methodological background and published examples of transparent reporting. The STROBE checklist is best used in conjunction with this article (freely available on the Web sites of PLoS Medicine at http://www.plosmedicine.org/, Annals of Internal Medicine at http://www.annals.org/, and Epidemiology at http://www.epidem.com/). Information on the STROBE Initiative is available at www.strobe-statement.org.
